# Evidence on the best way to perform oral hygiene with chlorhexidine in critically ill patients: systematic review and meta-analysis

**DOI:** 10.1186/1753-6561-5-S6-P72

**Published:** 2011-06-29

**Authors:** JR Gnatta, IRS Cabral de Menezes, RA Lacerda

**Affiliations:** 1 Nursing School, University of Sao Paulo, Sao Paulo, Brazil; 2 Comission Control Hospital Infection , University Hospital HU-USP, Sao Paulo, Brazil

## Introduction / objectives

Although the scientific literature has demonstrated the relevance of oral hygiene with chlorhexidine (CHX) in the prevention of pneumonia, there is wide variation in the product, concentration, frequency, duration and technique application.

## Methods

Systematic review and meta-analysis of articles in English, Spanish or Portuguese. Bases: Cochrane, EMBASE, Lilacs, PubMed/Medline, Ovid. Search: October to November 2010 using descriptors indexed. Question: *are there evidence about the best way to perform oral hygiene with chlorhexidinefor prevention of respiratory infection in critically ill patients on mechanical ventilation?*

## Results

10 primary studies were grouped in 4 groups (G1-4) based in criteria of concentration of CHX. G1 (10 primary studies with different concentrations of CHX) studies were homogeneous (Cochrane Q het p=0.35) and the common RR was significative (p<0.001 and CI=95%); G2 (5 primary studies CHX 0.12%) showed homogeneity (Cochrane Q het p=0.67) and the use of CHX represented protection (p<0.05); The G3 (3 primary studies CHX 0.2%) there was heterogeneity between studies (p=0.037) and CHX not represent a protective factor (p>0.05), G4 (2 primary studies CHX 2%) homogeneous studies (Cochran Q het p=0.62) and use of CHX was significant (p=0.021<0.05).

## Conclusion

If seems no doubt about the protective effect of oral hygiene with CHX in preventing pneumonia in critically ill patients, there is no evidence for adoption of protocols to guide the CHX concentration, as well as duration, frequency and application technique.

## Disclosure of interest

None declared.

